# Update of the MSKCC nomogram for metastatic progression and its role in active surveillance: the Italian TPCP cohort

**DOI:** 10.3389/fonc.2026.1799343

**Published:** 2026-05-13

**Authors:** Nicolas Destefanis, Daniela Zugna, Valentina Fiano, Renata Zelic, Michelangelo Fiorentino, Francesca Giunchi, Piero Fariselli, Mauro Giulio Papotti, Paola Cassoni, Marco Oderda, Paolo Gontero, Luca Lianas, Mauro Del Rio, Giuseppe Carlo Iorio, Umberto Ricardi, Olof Akre, Andreas Pettersson, Lorenzo Richiardi

**Affiliations:** 1 Cancer Epidemiology Unit, Department of Medical Sciences, University of Turin, Turin, Italy; 2 Department of Molecular Medicine and Surgery, Karolinska Institutet, Stockholm, Sweden; 3 Department of Pelvic Cancer, Cancer Theme, Karolinska University Hospital, Stockholm, Sweden; 4 DIMEC Department of Medicine and Surgery, Alma Mater Studiorum, University of Bologna, Bologna, Italy; 5 Department of Pathology, IRCCS Azienda Ospedaliero-Universitaria di Bologna, Bologna, Italy; 6 Computational Biomedicine Unit, Department of Medical Sciences, University of Turin, Turin, Italy; 7 Division of Pathology, University Hospital “Città della Salute e della Scienza di Torino” and University of Turin, Turin, Italy; 8 Department of Surgical Sciences, Urology Unit, Molinette Hospital, University of Turin, Turin, Italy; 9 Visual and Data-intensive Computing, CRS4 (Center for Advanced Studies, Research and Development in Sardinia), Pula, Italy; 10 Department of Oncology, Radiation Oncology, University of Turin, Turin, Italy; 11 Clinical Epidemiology Division, Department of Medicine Solna, Karolinska Institutet, Stockholm, Sweden; 12 Cancer Epidemiology Unit, University Hospital Città della Salute e della Scienza di Torino and CPO-Piemonte, Turin, Italy

**Keywords:** active surveillance, metastatic prostate cancer, prognosis, prognostic modelling, prostate cancer

## Abstract

**Background:**

Prognostic models are crucial for prostate cancer (PCa) treatment decision making at the time of diagnosis, particularly for distinguishing active surveillance (AS) candidates from those requiring curative treatment. While several models exist, their ability to predict metastatic disease—the primary driver of PCa mortality—remains underexplored.

**Methods:**

We analysed the Turin Prostate Cancer Prognostication cohort, which includes 891 unselected PCa patients diagnosed between 2008 and 2013 in Turin, Italy. Three widely used prognostic models—D’Amico, CAPRA, and MSKCC—were updated and compared based on optimism-corrected discrimination and overall prediction error for metastatic PCa (mPCa) within five years of diagnosis, accounting for competing risks. Overall survival was also assessed. Additionally, we investigated whether replacing standard AS eligibility criteria with nomogram-based risk thresholds could better identify patients at low risk of metastasis, maximizing AS uptake while minimising metastatic risk.

**Results:**

The MSKCC nomogram (optimism-corrected AUC*_t_*: 0.81; scaled Brier score: 0.15) outperforming the CAPRA score (AUC*_t_*: 0.77; Brier score: 0.11) and the D’Amico classification (AUC*_t_* 0.64; Brier score: 0.03) in predicting mPCa. The same ranking was observed for overall mortality prediction. When 95th percentile of MSKCC’s predicted probabilities among patients selected for six different AS protocols was used as a threshold, the proportion of potentially eligible patients increased from 7.8% when UCSF criterion was used to 57.0% without substantially increasing metastatic risk (observed 5-year risk: 1.7%).

**Conclusions:**

The MSKCC nomogram outperformed other models in predicting mPCa and overall mortality. Implementing risk-based AS eligibility thresholds derived from MSKCC could enhance patient selection while facilitating shared decision-making between patients and clinicians.

## 1 Introduction

Prostate cancer (PCa) was the most common cancer among men in Europe in 2022, accounting for 23% of all new cancer cases (excluding non-melanoma skin cancers) diagnosed in men, and the third leading cause of cancer death in men (1). PCa is a highly heterogeneous cancer in terms of prognosis. While metastatic PCa (mPCa) causes over 400,000 deaths each year, many men experience long-term treatment-related complications (2). Patients diagnosed with PCa have varying treatment options, determined by several clinical and pathological factors, including prostate-specific antigen (PSA) levels, clinical tumour stage, and the International Society of Urological Pathology (ISUP) grade group. These parameters guide the decision between active surveillance (AS) for less aggressive diseases, and radical treatment with surgery and/or radiotherapy. Patients with mPCa or limited life expectancy typically receive conservative or palliative care.

Prognostic models can provide useful guidance to treatment decisions at the time of diagnosis. The D’Amico classification system (3) and similar risk grouping tools are still widely used for pre-treatment risk assessment, but it is somewhat outdated. Even though several extended versions of the D’Amico groups have been proposed (4, 5), these still have some shortcomings: they overweight the importance of clinical stage, inconsistently consider the extent of biopsy involvement, do not consider age and other adverse parameters, and are unable to predict risk on a continuous scale, which offers greater precision and accuracy than grouping patients into broad risk categories (6, 7).

While previous studies have compared prognostic models head-to-head in terms of their ability to predict biochemical recurrence or PCa-specific mortality (8–12), none, to our knowledge, have focused on metastatic disease as the primary outcome. Prioritising the prediction of metastases could have significant implications, as metastatic disease is clinically distinct from biomedical recurrence and metastases are critical precursor to cancer-specific death (13), particularly among patients diagnosed after the age of 50 (14).

This study has two objectives. First, to update and compare, head-to-head, several pre-treatment tools of varying complexity (the D’Amico classification, the Cancer of the Prostate Risk Assessment (CAPRA) score (15), and Memorial Sloan Kettering Cancer Center (MSKCC) nomogram (16)) for prediction of mPCa in the Italian context using the Turin Prostate Cancer Prognostication (TPCP) cohort (17). Second, to evaluate the potential impact of using a nomogram-based model to select patients eligible for AS instead of basing the selection on pre-defined risk-based protocols, as it is current practice.

## 2 Methods

### 2.1 Study population

The TPCP cohort is a cohort of 891 consecutive patients diagnosed with PCa from 1st January 2008 to 31st December 2013, with a prostate biopsy evaluated at one of the two Pathology Divisions of the “A.O.U. Città della Salute e della Scienza di Torino,” Turin, Italy (hereafter referred to as “University Hospital”). A detailed description of the cohort has been published before (17). To be eligible, patients had to be under 85 years of age and without systemic metastases (MX or M0) at diagnosis–based on the available clinical data from the hospital medical charts, pathology reports, and imaging reports. All biopsy slides positive for PCa were digitised and centrally reviewed by the two study uropathologists using a digital pathology platform (18) to assign ISUP grade group and other histopathological characteristics at the slide, core, or cancer tissue level. For each patient, we calculated the Charlson-Romano Comorbidity Index (CRCI) (19) using information on all hospital admissions at the University Hospital over the five years before the diagnosis of PCa. Finally, each patient was assigned a Social Deprivation Index (SDI) value, based on databases available for the whole country at the census level (20).

The primary outcome was the occurrence of mPCa, defined as the first instance of metastatic disease following diagnosis at the University Hospital medical charts. The event date is recorded as the date when the metastasis was initially detected. Assuming that lethal PCa always progresses through a metastatic stage, for 13 patients who died from PCa but did not have a record of metastasis, the date was imputed as previously described (17). The secondary outcome was overall mortality. Each patient was followed from the date of the diagnostic biopsy report until the date of metastases, death, emigration outside the Province of Turin, or 31st December 2021, whichever came first. Life status was verified using demographic records from various municipalities, while the specific cause of death was determined from death certificates provided by local health authorities and categorised as either PCa-specific death or death from other causes.

The TPCP cohort study was approved by the Ethical Committee of the “Comitato Etico Interaziendale A.O.U. Città della Salute e della Scienza di Torino” (approval number 595/2020).

### 2.2 Statistical analyses

#### 2.2.1 Missing data

Around 20% of patients had missing values in at least one of the predictors. The proportion of missing data ranged from 0.9% for ISUP grade group to 7.3% for clinical tumour stage (**Table 1**). We assumed the data were missing at random and used multiple imputation with chained equations to impute 20 datasets (21). For the primary clinical tumour stage, the ISUP grade group, PSA and number of positive cores, we used the predictive mean matching method. For the clinical tumour substages, we used a conditional imputation based on the value of the primary tumour stage. Finally, a post-processing rule was implemented to ensure that the imputed number of positive biopsy cores did not exceed the total number of biopsy cores. The Nelson-Aalen estimator of the cumulative hazard was computed separately for metastatic PCa and death from other causes, and both were included as predictors in all imputation models alongside the respective event status indicators. All estimates were combined according to Rubin’s rules (22).

**Table 1:** Baseline characteristics of the predictors in the TPCP cohort.

Variable	TPCP Cohort	
	*n =* 891	%
Age at Diagnosis (years)		
Median (IQR)	69 (64–74)	–
PSA^a^ (ng/mL)		
Median (IQR)	6.7 (5.0, 10.0)	–
Missing	23	–
ISUP^b^ grade group		
1	124	14.0
2	287	32.5
3	165	18.7
4	225	25.5
5	82	9.3
Not evaluable^c^	8	–
Main cT		
cT1	366	44.5
cT2	288	35.0
cT3	169	20.5
Missing	68	–
Detailed cT		
cT1c	366	44.5
cT2a	106	12.9
cT2b	39	4.7
cT2ab^d^	39	4.7
cT2c	104	12.6
cT3+	169	20.5
Missing	68	–
Number of positive cores sampled at biopsy		
Median (IQR)	3.0 (1.0, 5.0)	–
Missing	56	–
Number of negative cores sampled at biopsy		
Median (IQR)	11.0 (8.0, 14.0)	–
Missing	56	–
Charlson-Romano Comorbidity Index		
0	447	50.2
1	36	4.0
2	51	5.7
3+	35	3.9
Without admissions in the previous 5 years	322	36.1
Social Deprivation Index^e^		
Low	280	31.4
Medium	280	31.4
High	331	37.1
Treatment (within six months)		
Prostatectomy	362	40.6
Radiotherapy	137	15.4
Androgen Deprivation Therapy	124	13.9
Deferred^f^	372	41.8

IQR, Interquartile Range (25^th^–75^th^ percentile).

a PSA, Prostate-Specific Antigen.

b ISUP, International Society of Urological Pathology.

c Gleason score for patients with a tumour area of less than 5% was not evaluable.

d Patients with cT2ab clinical stage were classified as missing.

e The Social Deprivation Index variable was categorised into tertiles based on the distribution among the TPCP cohort members.

f Deferred treatment includes active surveillance or watchful waiting.

#### 2.2.2 Update of the models

Within a competing-risk framework, we used cause-specific Cox proportional hazards models to estimate cause-specific hazards of mPCa and the death from causes different from PCa. The estimates obtained from the two Cox models were then combined to estimate the cumulative incidence curves.

When modelling mPCa, for the D’Amico classification and the CAPRA score we assigned each patient to the corresponding category/score, which was then modelled as a single covariate, while for MSKCC nomogram, each potential predictor was included separately (**Supplementary Table 1**). For MSKCC, PSA at diagnosis was included using restricted cubic splines (RCSs) with four knots (at 5th, 35th, 65th, and 95th percentile). For models predicting death from other causes, we included age at diagnosis in the D’Amico classification (as it does not account for age) and used the CRCI and SDI for all models, with SDI modelled using RCSs with four knots. The same variables were used for overall mortality. A Cox proportional hazards model was used for overall mortality.

#### 2.2.3 Validation and head-to-head comparison

We performed internal validation using bootstrapping (n=500). Discrimination was evaluated in terms of optimism-corrected (23) cumulative-dynamic area under the receiving operator characteristic curve (AUC*_t_*) (24), while the overall error prediction was measured by the scaled Brier score (25). Calibration was evaluated by estimating the optimism-corrected calibration intercept and slope and by plotting observed versus predicted probabilities of the outcome at five years (26, 27). The observed outcome probabilities were estimated using pseudo-values (28). For each of the 20 imputed datasets, we pooled results by taking the median across the 500 bootstrapped samples. Finally, the medians of these pooled values were averaged across imputations.

The updated models were compared based on the discrimination and overall prediction error for the development of metastatic disease at five years post-diagnosis. The model with the highest AUC*_t_* and the highest scaled Brier score was considered the best performing model. The calibration plot is shown only for the best performing model.

#### 2.2.4 Impact on selection of patients for active surveillance

The best performing model was used to identify patients potentially eligible for AS in comparison with AS selection protocols commonly used in the clinical routine (29), including Miami (30), MSKCC (31), Royal Marsden (32), Sunnybrook (33), and University of California and San Francisco (UCSF) (34). We also included one protocol that is currently implemented among the University Hospital patients (35). We did not consider AS protocols that require PSA density, as this variable was not available in the TPCP cohort.

For each protocol, we applied the inclusion criteria (**Supplementary Table 2**) to the TPCP cohort to identify potentially eligible patients. We then determined the 95th percentile of the distribution of the five-year probability of developing mPCa predicted using the best performing model among the identified eligible patients. Then, the overall, arbitrary, threshold for selection for AS was obtained by averaging the protocol-specific 95th percentiles over the different AS protocols. Finally, for each protocol and for the chosen threshold, we calculated the number of patients potentially eligible for AS and their five-year non-parametric cumulative incidence of PCa metastases. The best preferred approach is the one that at the same time maximises the number of patients who are selected for AS and minimises their risk of developing mPCa. To illustrate the effect of the threshold choice on AS eligibility, we additionally evaluated three threshold scenarios: 3%, 4%, and 5%.

## 3 Results

The TPCP cohort was followed-up for a median duration of 10 years (IQR: 8.2–12.9) and a maximum duration of 14 years, during which 97 (10.9%) patients developed a metastatic disease, and 301 (33.8%) patients died; of these, 56 (6.3%) died from PCa. The distribution of key variables of the cohort is provided in **Table 2**.

**Table 2:** Bootstrap internal validation of performance measures (AUC*_t_*, Brier score, calibration intercept and calibration slope) of the models, predicting the occurrence of metastatic prostate cancer and overall mortality within five years post-diagnosis.

Variable	Measure	Model	Apparent (95% CI)	Optimism	Optimism-corrected
Metastatic Prostate Cancer	AUC*_t_*	MSKCC	0.83 (0.78, 0.90)	0.02	0.81
		CAPRA	0.77 (0.69, 0.85)	0.00	0.77
		D’Amico	0.64 (0.56, 0.71)	0.00	0.64
	Brier Score	MSKCC	0.19 (0.11, 0.32)	0.04	0.15
		CAPRA	0.12 (0.05, 0.21)	0.00	0.11
		D’Amico	0.03 (0.01, 0.05)	0.00	0.03
	Intercept	MSKCC	0.03 (-0.05, 0.21)	0.07	-0.04
		CAPRA	0.04 (-0.06, 0.19)	0.01	0.04
		D’Amico	-0.01 (-0.06, 0.07)	0.00	-0.01
	Slope	MSKCC	1.09 (0.95, 1.83)	0.12	0.98
		CAPRA	1.07 (0.84, 1.48)	0.01	1.06
		D’Amico	0.98 (0.69, 1.90)	0.02	0.97
Overall Mortality	AUC*_t_*	MSKCC	0.80 (0.76, 0.85)	0.02	0.78
		CAPRA	0.74 (0.69, 0.80)	0.01	0.74
		D’Amico	0.76 (0.71, 0.81)	0.01	0.76
	Brier Score	MSKCC	0.18 (0.12, 0.27)	0.03	0.15
		CAPRA	0.10 (0.06, 0.16)	0.01	0.09
		D’Amico	0.13 (0.08, 0.21)	0.01	0.12
	Intercept	MSKCC	0.04 (-0.01, 0.16)	0.04	-0.0
		CAPRA	0.04 (0.02, 0.12)	0.01	0.03
		D’Amico	0.03 (-0.03, 0.12)	0.01	0.02
	Slope	MSKCC	1.04 (0.85, 1.31)	0.10	0.94
		CAPRA	1.05 (0.93, 1.39)	0.05	1.00
		D’Amico	1.08 (0.80, 1.42)	0.04	1.04

*MSKCC*, Memorial Sloan Kettering Cancer Centre.

*CAPRA*, Cancer of the Prostate Risk Assessment.

### 3.1 Update of the models and head-to-head comparison

Based on the pooled optimism-corrected time-dependent AUC*_t_* and scaled Brier score for the model predicting five-year mPCa, the D’Amico classification performed the worst (AUC*_t_* 0.64, Brier score 0.03), followed by the CAPRA score (AUC*_t_* 0.77, Brier score 0.11), while the MSKCC was identified as the top performer (AUC*_t_* 0.81, Brier score 0.15) (**Table 2**). There was no change in the order of performance over follow-up time (**Supplementary Figure 3**).

Overall, at five years, models predicting overall mortality performed similarly to the models predicting mPCa, with only a minor decrease in AUC*_t_* and a negligible increase in the scaled Brier scores (**Table 2**), except for the D’Amico classification. For the latter, both the discrimination (AUC*_t_* 0.76) and the overall error (Brier score 0.12) improved likely due to the inclusion of continuous age in the model. The performance decay for CAPRA score, which includes only dichotomised age, and performance improvement for D’Amico classification were more prominent at later follow-up times (**Supplementary Figure 3**).

For all three models and most of the model performance measures, except calibration slope, there was little optimism in the apparent estimates of performance at five years (**Table 2**; **Supplementary Figures 1**, **2**) and over entire follow-up time (**Supplementary Figure 3**).

### 3.2 Calibration of the updated MSKCC nomogram (MSKCC_ITA)

**Figure 1:**
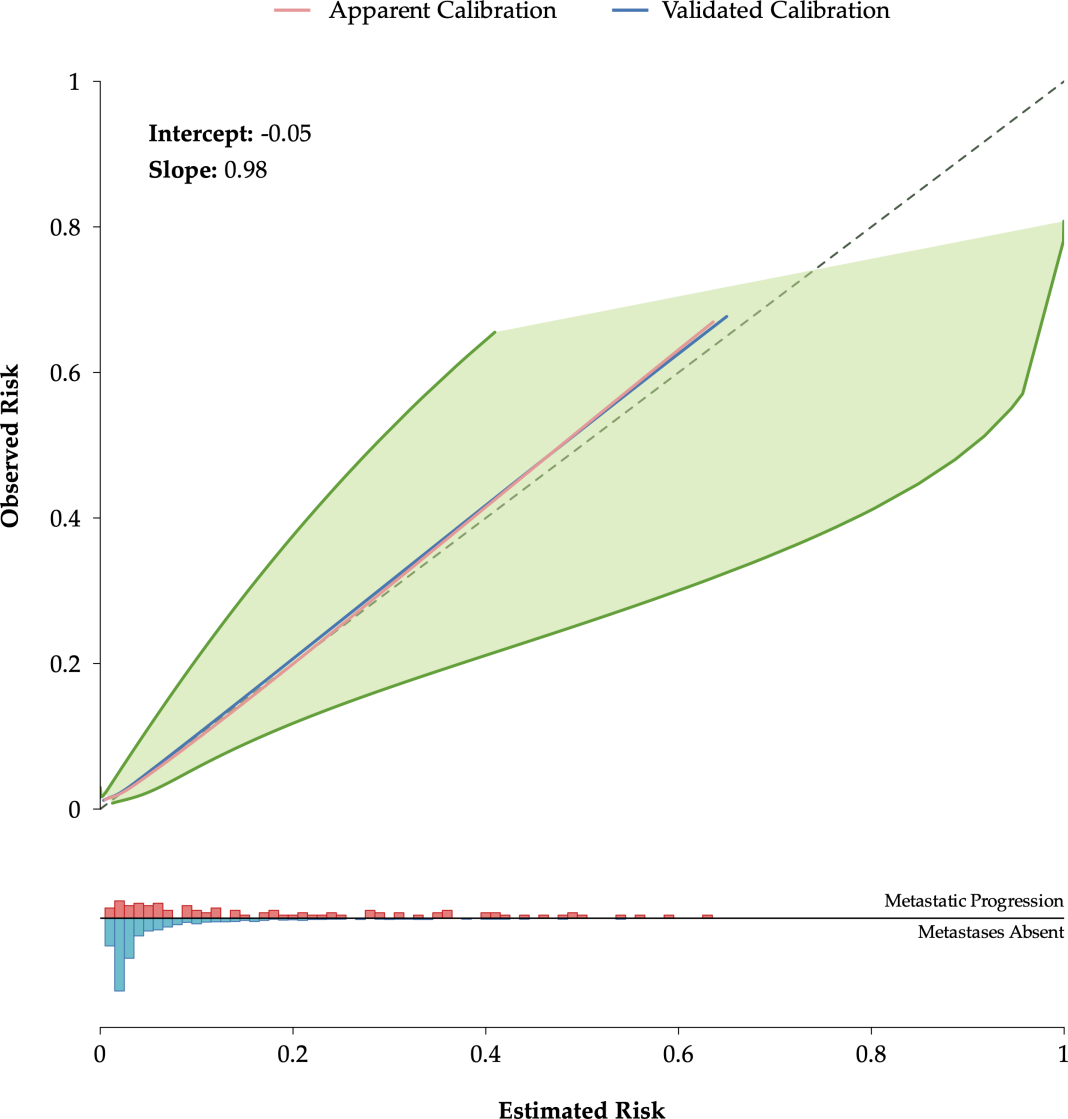
MSKCC calibration plot for metastatic prostate cancer at five years post-diagnosis, following internal validation using bootstrap sampling (500 repetitions) and multiple imputation across 20 datasets. The “apparent” and “validated” lines represent the median calibration plots across the 20 imputed datasets. Confidence intervals are defined by the medians of the 2.5th and 97.5th percentiles across the 500 bootstrap samples for each imputed dataset. The histogram along the x-axis shows the distribution of risk estimates, stratified by metastatic and non-metastatic patients. MSKCC, Memorial Sloan Kettering Cancer Center.

Pooled coefficients, HRs, and corresponding 95% CIs for the MSKCC_ITA models predicting mPCa and overall mortality across the 20 imputed datasets are reported in **Supplementary Table 3**. In addition to calibration intercept and slope, the calibration of the MSKCC_ITA model was assessed by plotting the five-year probabilities of mPCa and overall mortality pooled across 500 bootstraps and across the 20 imputed datasets. For the MSKCC_ITA model predicting five-year probabilities of mPCa, the optimism corrected intercept was close to 0 (-0.04), the slope was close to 1 (0.98) and the predicted and the observed probabilities were similar, indicating overall good calibration and little optimism (**Figure 1**, **Supplementary Figure 4**). A zoomed calibration plot for the 0-0.15 risk range is provided in **Supplementary Figure 5**. For the MSKCC_ITA model predicting five-year overall mortality, the calibration intercept was 0 (0.00), the slope was close to 1 (0.94), but plot indicates overestimation of higher and lower probabilities and underestimation of middle-range probabilities, but with no optimism in the estimates (**Supplementary Figures 6**, **7**).

### 3.3 Implications of a nomogram-based approach for the selection of patients for active surveillance

The number of patients potentially eligible for AS varied widely across the six examined protocols, ranging from 56 (7.8%) to 180 (25.1%) out of 716 patients (**Table 3**). This analysis was restricted to the 716 patients with complete data on all variables required to apply the AS protocols, as the application of eligibility criteria requires observed rather than imputed values.

**Table 3:** Comparison of standard active surveillance protocols with a nomogram-based approach based on the number of eligible patients, observed metastatic cases at five years, and corresponding metastatic risk.

Active surveillance protocol	Eligible patients	Metastatic individuals among eligible patients (five years)	5-year risk^a^ (%)	Metastatic individuals among eligible patients (ten years)	10-year risk^a^ (%)	MSKCC 95^th^ risk percentile among eligible patients^b^ (%)
Miami (1992) (30)	62	1	1.61	2	3.23	4.47
MSKCC (1997) (31)	57	2	3.51	3	5.77	3.71
Royal Marsden (2002) (32)	180	5	2.78	10	5.68	3.76
Sunnybrook (2015) (33)	150	5	3.33	8	5.47	4.47
UCSF (1990) (34)	56	1	1.79	2	4.08	3.66
START (2023) (35)	75	3	4.00	4	5.62	3.59
Nomogram-based	408	7	1.72	15	3.73	3.94^c^

a Percentage of metastatic patients among those eligible for the active surveillance protocol. We evaluated the five- and ten-year non-parametric cumulative incidence after diagnosis to account for the presence of competing risks.

b We calculated the individual probabilities of metastatic prostate cancer occurrence at five years post-diagnosis using the MSKCC pre-treatment nomogram. For each active surveillance protocol, we selected the 95^th^ percentile of these probabilities as the threshold for directing patients.

c This threshold, used to include patients in a hypothetical active surveillance protocol, was determined by taking the mean of the predicted probabilities above the 95^th^ percentile for the mentioned protocols.

*MSKCC*, Memorial Sloan Kettering Cancer Centre.

*UCSF*, University of California and San Francisco.

*START*, “Sorveglianza attiva o Trattamento Radicale alla diagnosi per Tumori della prostata a basso rischio”.

**Figure 2:**
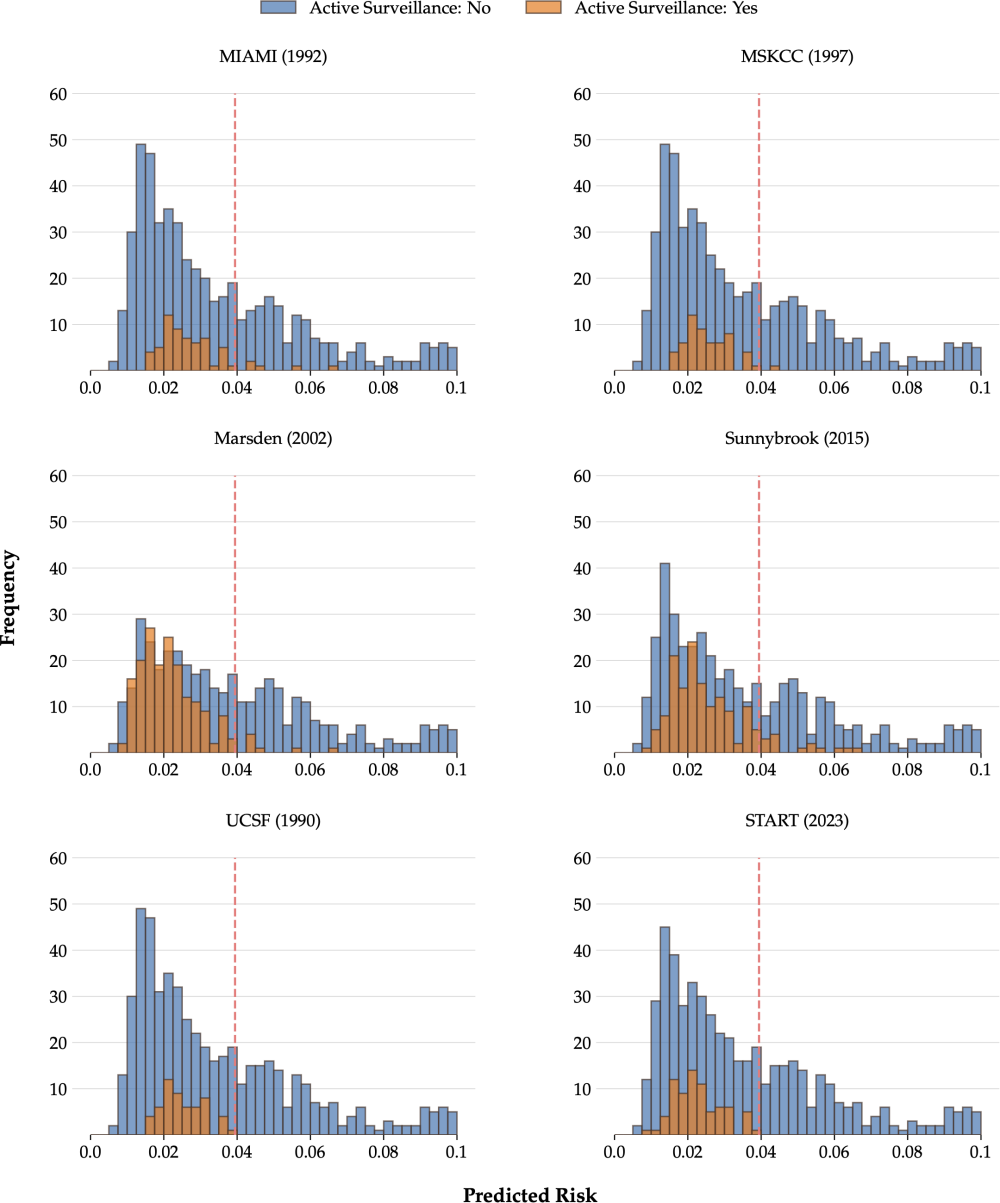
Histograms of MKSCC’s predicted five-year risk of metastatic prostate cancer for patients potentially included (orange) or not (blue) in six different active surveillance protocols. Risk was truncated at 0.10. Dashed-line indicates the arbitrary threshold that could be for the selection of patients for active surveillance. MSKCC, Memorial Sloan Kettering Cancer Center; UCSF, University of California and San Francisco.

Likewise, the observed five-year risk of mPCa varied from 1.6% (Miami protocol, based on 62 patients) to 4.0% (START protocol, based on 75 patients). When potential eligibility for AS was instead determined using the MSKCC_ITA nomogram with an arbitrary threshold of 3.9% (see Methods), the number of potentially eligible patients increased substantially to 408 (60.0%), while the observed five-year risk of mPCa remained low (1.7%). When we look at the distribution of MSKCC_ITA’s predicted five-year risk of mPCa among patients who would have been included or excluded according to the criteria of the respective AS protocol, the majority of patients with low predicted risk of metastasis would have been excluded due to the categorical nature of the protocols, which rely on dichotomous criteria (**Figure 2**, **Supplementary Table 2**). Calibration in this low-risk range was confirmed to be accurate (**Supplementary Figure 5**).

In the scenario analysis, the proportion of eligible patients ranged from 46% (329 out of 716) at the 3% threshold to 66% (470 out of 716) at the 5% threshold, with observed 5- and 10-year metastatic risks of 1.52%, 3.70% and 2.55%, 5.40%, respectively. At the 4% threshold, 413 patients (58%) were eligible with observed 5- and 10-year risks of 1.69% and 3.70%. The probability of never developing metastatic progression over the entire follow-up was 95.8% and 95.9% at the 3% and 4% thresholds, respectively, dropping to 93.2% at the 5% threshold (**Table 4**). Calibration in this low-risk range was confirmed to be accurate (**Supplementary Figure 5**).

**Table 4:** Analysis of different scenarios evaluating three nomogram-based risk thresholds for active surveillance eligibility, applied to the 716 patients with complete data in the TPCP cohort.

Risk threshold	Eligible patients	Metastatic individuals among eligible patients (5 years)	5-year risk^a^ (%)	Metastatic individuals among eligible patients (10 years)	10-year risk^a^ (%)	Never developing mPCa^b^ (%)
3%	329	5	1.52	12	3.71	95.8
4%	413	7	1.69	15	3.68	95.9
5%	470	12	2.55	25	5.37	93.2

a Percentage of metastatic patients among those eligible for the active surveillance protocol. We evaluated the five-year non-parametric cumulative incidence after diagnosis to account for the presence of competing risks.

b Probability estimated as 1 minus the cumulative incidence at the maximum observed follow-up time using the Aalen-Johansen estimator, accounting for competing risks.

## 4 Discussion

In this study, we evaluated several prognostic models for PCa in Italy, focusing on metastatic disease as the primary outcome, and evaluated the impact of using a nomogram for AS selection. The MSKCC_ITA nomogram performed best, suggesting that models which use full information contained in continuous variables without forced linear associations provide superior predictive accuracy than category-based models.

There is an ongoing debate about how to safely expand the criteria for case selection to include more patients in AS (36–38). Given the significant side effects associated with curative-intent treatments, AS has recently been suggested for select men with ISUP grade group 2 disease (29). Currently, all AS protocols rely on risk group categorisation to determine eligibility (2, 30, 39–42). However, substantial heterogeneity exists within risk groups, particularly among men traditionally considered ineligible for AS. An alternative approach is to base AS eligibility on individual absolute risk predictions rather than broad risk categories. In this study, we found that an individualised risk-based approach could increase the number of men eligible for AS while maintaining acceptably low risks of metastasis. The 3.9% threshold should not be interpreted as a fixed clinical recommendation, but rather as an illustration of how nomogram-based risk stratification could support individualised decision-making. We therefore advocate for transitioning to a system that prioritizes individual absolute risk predictions over rigid risk group classifications. To support this shift and facilitate clinical decision-making, we plan to develop an online version of the MSKCC_ITA calculator, making it easily accessible to clinicians and researchers. However, to prevent the underestimation of disease progression risk during AS, incorporating novel biomarkers would be beneficial, as traditional clinical parameters remain insufficient. Formal threshold selection (incorporating competing mortality and treatment-related morbidity) would require a dedicated decision analysis, which is beyond the scope of the current study and represents a natural next step.

Our study has several strengths. First, while some previous studies have compared prognostic models, none, to our knowledge, have specifically focused on predicting metastatic progression–a critical event in PCa that significantly impacts survival, quality of life, and treatment decisions. Second, we addressed missing data through imputation, ensuring more robust and complete analyses. Third, to mitigate overfitting, all performance measures were optimism-corrected, preventing an overly favourable impression of model performance.

This work has some limitations, the most notable being that the cohort includes patients diagnosed before 2015 to ensure a long duration of follow-up, which is prior to the widespread adoption of multi-parametric magnetic resonance imaging (mpMRI). This impacts the number and type of patients undergoing biopsy for suspected PCa. It is important to note, however, that the primary clinical benefit of mpMRI is the reduction of unnecessary biopsies of benign prostate tissue or indolent tumours, most of which should be classified as low risk by the discussed models. Therefore, while MRI-guided diagnostics may alter pre-treatment risk group assignments, the overall prognostic impact remains modest in men referred from unscreened populations (43). Finally, the AS analysis was based on a 5-year prediction horizon, which underestimates long-term metastatic risk. However, the probability of never developing metastatic progression over the entire follow-up (up to approximately 10 years) remained high across all nomogram-based thresholds (93-96%, **Table 4**), providing some reassurance about the longer-term safety of this approach. Future studies with longer follow-up are needed to better inform clinical decision-making for younger patients with extended life expectancy.

## 5 Conclusions

We updated prognostic models for PCa to the Italian context, focusing on mPCa as the primary outcome. We also evaluated the potential impact of using MSKCC’s predicted probabilities to select patients for AS instead of standard risk-based protocols, which is the current standard in most urology departments. This approach could improve patient selection and support personalised, shared decision-making based on individualised risk.

## Data Availability

The data analyzed in this study is subject to the following licenses/restrictions: The datasets analysed during the current study are not publicly available due to legal and ethical restrictions. However, researchers interested in using the TPCP cohort data for replication or external validation of prognostic models for prostate cancer may contact Lorenzo Richiardi (lorenzo.richiardi@unito.it), Principal Investigator of the TPCP cohort, to discuss potential data access. While data sharing is limited under the current legal framework, we are open to supporting data reuse by reviewing proposed analysis plans, implementing them within our cohort, and sharing the resulting outputs. Requests to access these datasets should be directed to Lorenzo Richiardi, lorenzo.richiardi@unito.it.
