# Supplementary material for: Update of the MSKCC nomogram for metastatic progression and its role in active surveillance: the Italian TPCP cohort
Source: Front Oncol. 2026 May 13;16:1799343. doi: 10.3389/fonc.2026.1799343 (PMC13212092; doi:10.3389/fonc.2026.1799343)
Supplement: Supplementary file 1 [file DataSheet1.pdf]

Supplementary Material

1 SUPPLEMENTARY TABLES AND FIGURES

1.1 Tables

Table S1. Variables included in the D'Amico risk stratification tool, the CAPRA score, and MSKCC nomogram.

	**D'Amico**	**CAPRA**	**MSKCC**
Age	—	•	•
PSA	•	•	•
ISUP	•	•	•
cT	•	•	•
PBP	—	•	—
Number of positive cores	—	—	•
Number of negative cores	—	—	•

PSA: Prostate-specific antigen.
ISUP: International Society of Urological Pathology.
PBP: Percent Biopsy Positivity.
CAPRA: Cancer of the Prostate Risk Assessment.
MSKCC: Memorial Sloan Kettering Cancer Center.

Table S2. Active surveillance protocols' inclusion criteria.

Active Surveillance Protocol	Notes	cT Stage	ISUP Grade Group	PSA (ng/mL)	Number of Cores	Core Involvement (%)
MIAMI	—	cT1 – cT2	≤ 1	≤ 15	≤ 2	< 20
MSKCC	—	cT1c – cT2a	≤ 1	≤ 10	≤ 3	< 50
MARSDEN	Overall	cT1 – cT2	≤ 1	≤ 15	$< 50\%$ cores	—
	Age > 65		≤ 2			—
SUNNYBROOK	All low-risk	—	≤ 1	≤ 10	—	—
	Age > 70	—	≤ 2	≤ 15	—	—
UCSF	—	cT1c – cT2a	≤ 1	≤ 10	$< 33\%$ cores	< 50
START	Overall	cT1c – cT2a	≤ 1	≤ 10	Varies*	—
	Age > 70		≤ 2	≤ 10		—

cT: Clinical stage.
ISUP: International Society of Urological Pathology.
PSA: Prostate-specific antigen.
MSKCC: Memorial Sloan Kettering Cancer Center.
UCSF: University of California and San Francisco.
START: "Sorveglianza attiva o Trattamento Radicale alla diagnosi per Tumori della prostata a basso rischio".
*Maximum number of positive biopsy cores for prostate cancer:
- 2, in case of biopsy with random sampling and less than 20 samples;
- 3, in case of biopsy with random sampling and 21-25 samples;
- 4, in case of biopsy with random sampling and more than 26 samples.

Table S3: Pooled coefficients, hazard ratios and 95% confidence intervals from the cause-specific Cox proportional hazards models predicting metastatic prostate cancer and overall mortality across the 20 imputed datasets at five years after diagnosis for the MSKCC nomogram. Restricted cubic spline bases for PSA and SDI were orthogonalised prior to model fitting to ensure numerical stability. SDI (with their spline terms) and CRCI were included in the overall mortality model only.

	**Metastatic Prostate Cancer**			**Overall Mortality**		
**Variable**	**log(HR)**	**HR**	**95% CI**	**log(HR)**	**HR**	**95% CI**
Age at diagnosis (standardised)	0.13	1.14	0.91 – 1.43	0.77	2.15	1.87 – 2.48
PSA	0.23	1.26	1.10 – 1.44	0.18	1.20	1.09 – 1.32
PSA (RCS1)	-0.24	0.79	0.64 – 0.97	-0.15	0.86	0.78 – 0.96
PSA (RCS2)	0.02	1.02	0.80 – 1.30	-0.11	0.89	0.80 – 1.00
ISUP 1	*Ref.*	*Ref.*	–	*Ref.*	*Ref.*	–
ISUP 2	-0.48	0.62	0.21 – 1.88	0.21	1.24	0.78 – 1.96
ISUP 3	0.30	1.35	0.46 – 3.92	0.42	1.52	0.93 – 2.46
ISUP 4	0.93	2.53	0.94 – 6.78	0.35	1.41	0.88 – 2.28
ISUP 5	1.68	5.36	1.88 – 15.31	0.75	2.11	1.21 – 3.68
cT 1c	*Ref.*	*Ref.*	–	*Ref.*	*Ref.*	–
cT 2a	0.38	1.46	0.64 – 3.33	0.21	1.24	0.82 – 1.88
cT 2b	-0.30	0.74	0.19 – 2.83	-0.01	0.99	0.56 – 1.74
cT 2c	0.53	1.70	0.82 – 3.52	0.23	1.26	0.84 – 1.89
cT 3+	0.87	2.38	1.29 – 4.37	0.08	1.08	0.76 – 1.54
Number of positive cores	-0.01	0.99	0.90 – 1.08	-0.002	1.00	0.95 – 1.05

Continued on next page

	Metastatic Prostate Cancer			Overall Mortality		
**Variable**	**log(HR)**	**HR**	**95% CI**	**log(HR)**	**HR**	**95% CI**
Number of negative cores	-0.05	0.95	0.90 – 1.01	-0.02	0.98	0.95 – 1.01
SDI	–	–	–	0.09	1.10	0.96 – 1.25
SDI (RCS1)	–	–	–	-0.21	0.81	0.71 – 0.93
SDI (RCS2)	–	–	–	0.01	1.01	0.89 – 1.14
CRCI 0	–	–	–	*Ref.*	*Ref.*	–
CRCI 1	–	–	–	0.51	1.67	1.05 – 2.65
CRCI 2	–	–	–	0.89	2.43	1.79 – 3.32

MSKCC: Memorial Sloan Kettering Cancer Center.
HR: Hazard ratio.
CI: Confidence interval.
cT: Clinical stage.
PSA: Prostate-specific antigen.
RCS: Restricted cubic spline.
ISUP: International Society of Urological Pathology.
SDI: Social deprivation index.
CRCI: Charlson–Romano comorbidity index.

1.2 Figures

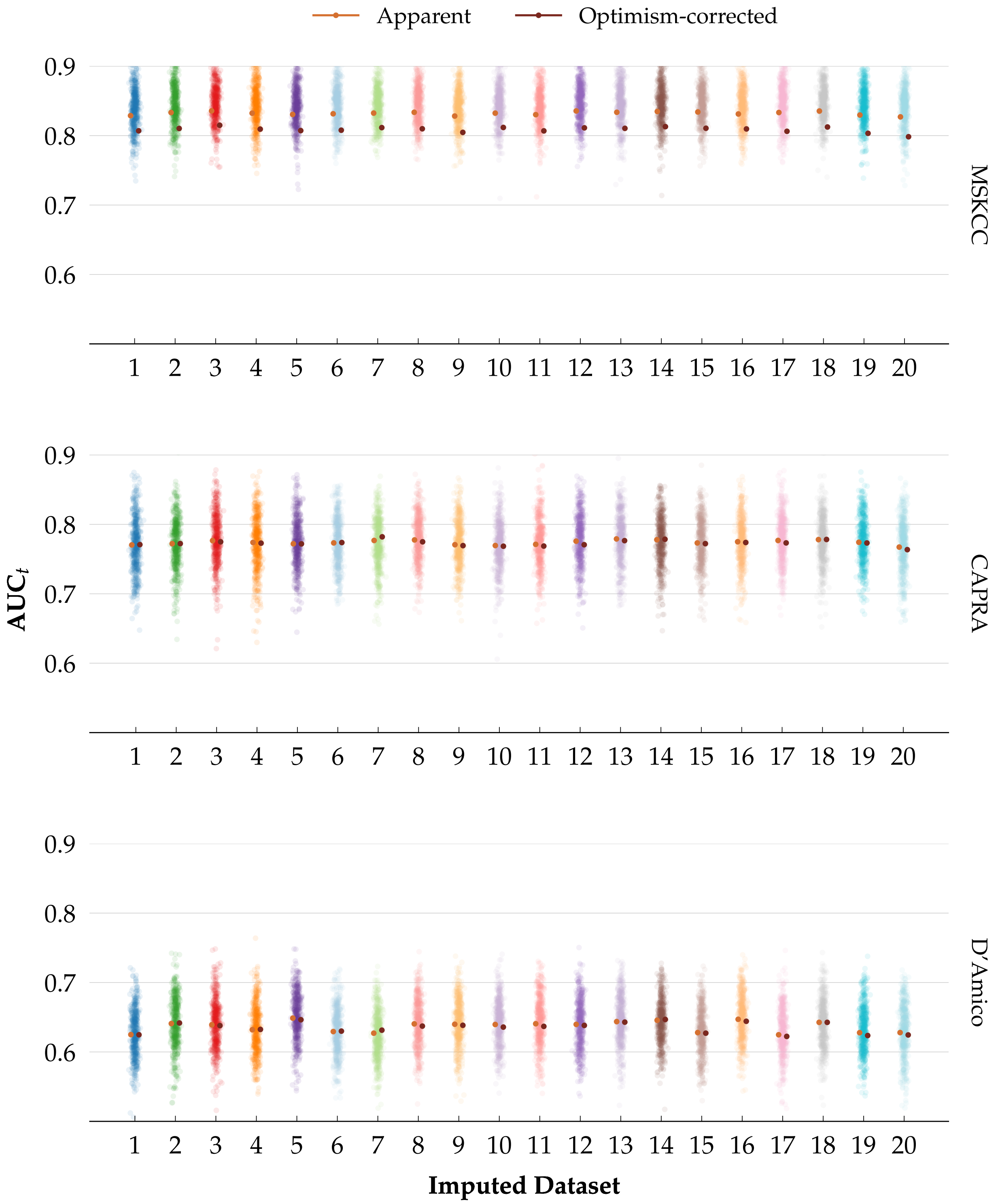

Figure S1: Comparison of AUC_t estimates (apparent and optimism-corrected) for various prognostic models for metastatic prostate cancer, evaluated over several imputed datasets at five years post-diagnosis. The distributions of bootstrap estimates across the imputed datasets are also shown. CAPRA: Cancer of the Prostate Risk Assessment; MSKCC: Memorial Sloan Kettering Cancer Centre.

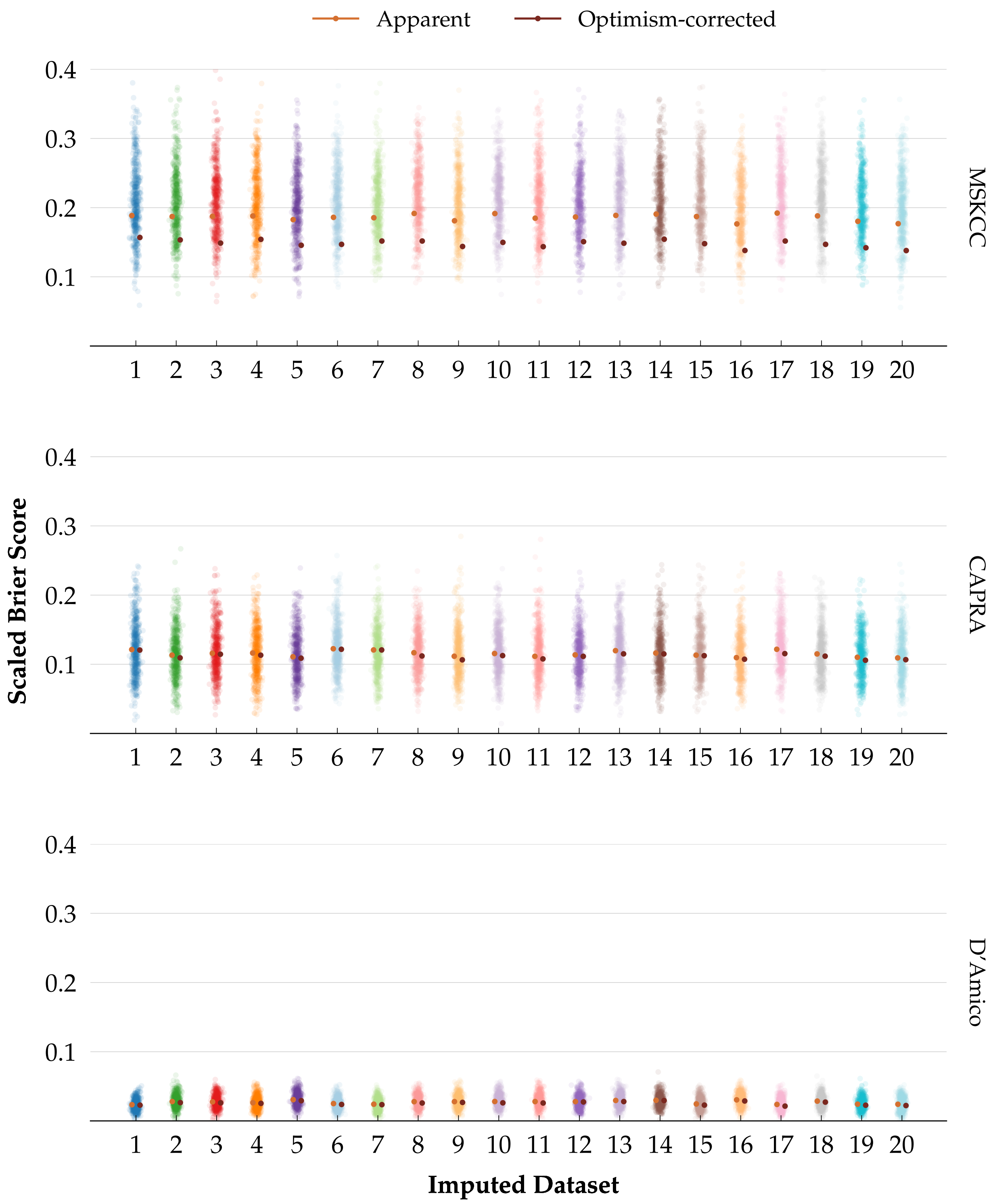

Figure S2: Comparison of scaled Brier score estimates (apparent and optimism-corrected) for various prognostic models for metastatic prostate cancer, evaluated over several imputed datasets at five years post-diagnosis. The distributions of bootstrap estimates across the imputed datasets are also shown. CAPRA: Cancer of the Prostate Risk Assessment; MSKCC: Memorial Sloan Kettering Cancer Centre.

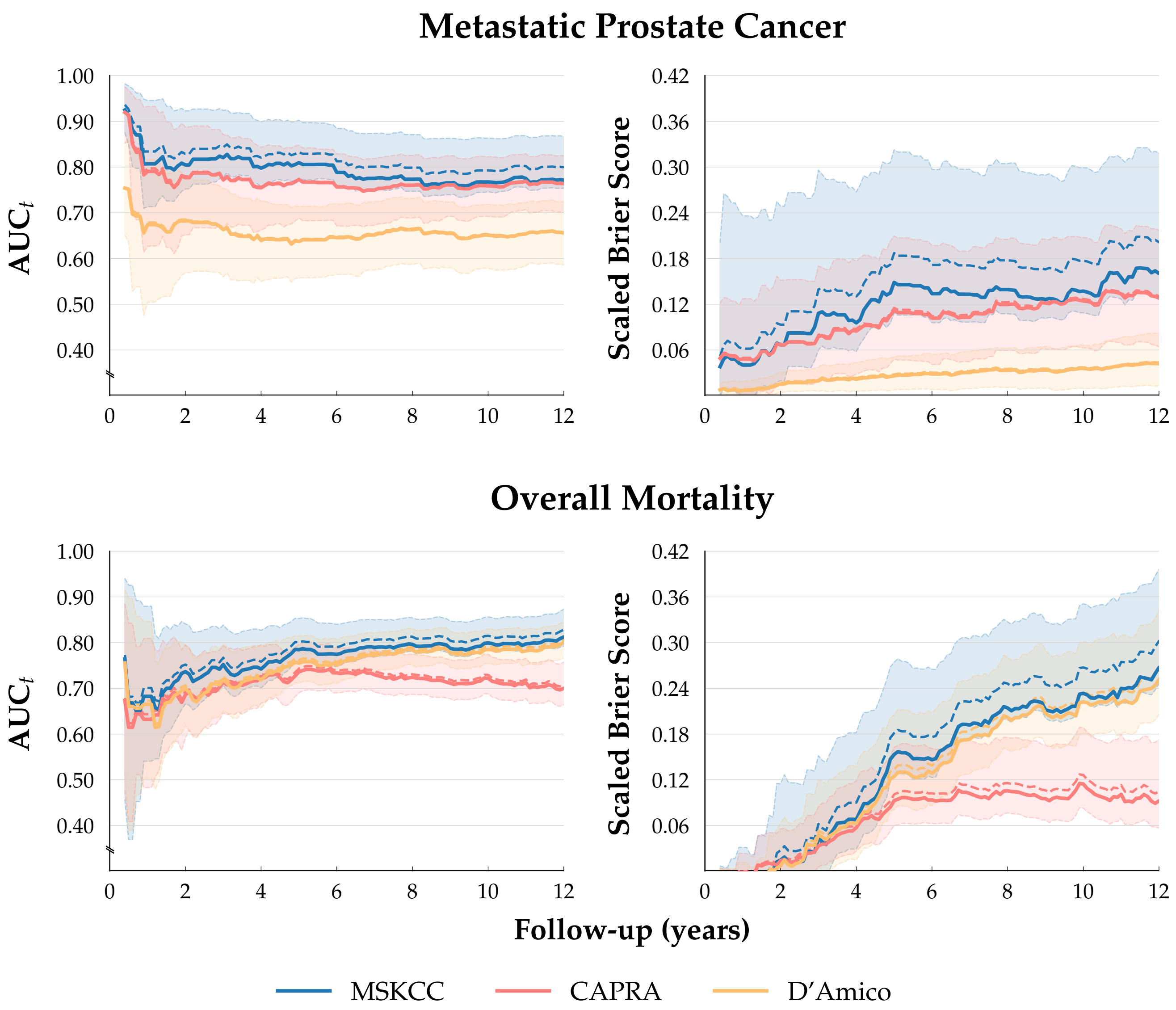

Figure S3: Apparent and optimism-corrected AUC_t and scaled Brier score for metastatic prostate cancer and overall mortality at five years after diagnosis, with 95% confidence intervals. The apparent (dashed line) and optimism-corrected (continuous line) estimates correspond to the median of the apparent estimates and the median of the optimism-corrected estimates, respectively. The confidence intervals are defined by the medians across the 20 imputed datasets of the 2.5th and 97.5th percentiles across the 500 bootstrap samples. CAPRA: Cancer of the Prostate Risk Assessment; MSKCC: Memorial Sloan Kettering Cancer Center.

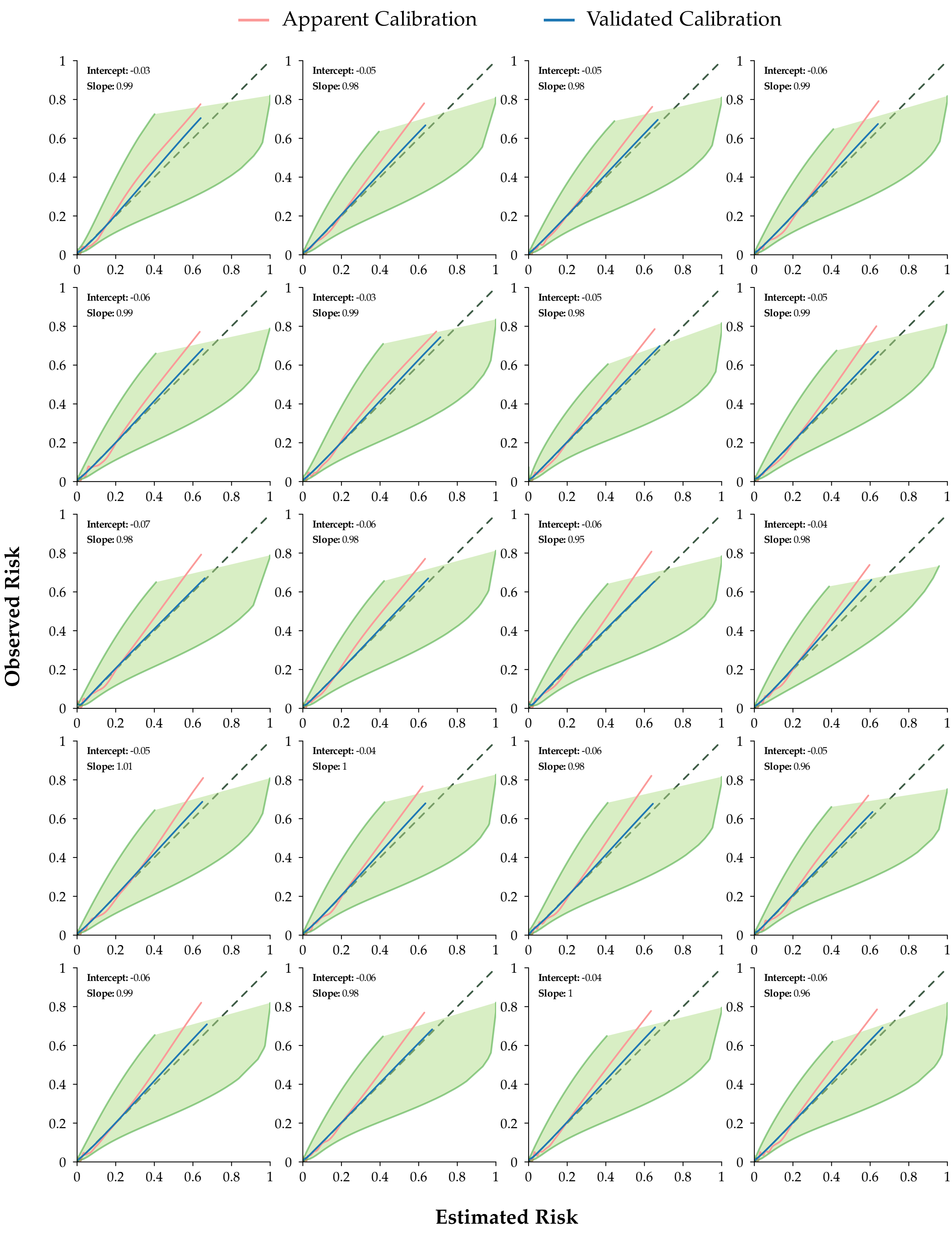

Figure S4: Apparent and internally validated calibration plots for MSKCC predicting the insurgence of metastatic prostate cancer five years after diagnosis, for each imputed dataset. The green area represents the range between the 2.5th and 97.5th percentiles across the 500 bootstrap samples. Intercept and slope estimates for the outcome are reported for each imputed dataset. MSKCC: Memorial Sloan Kettering Cancer Center.

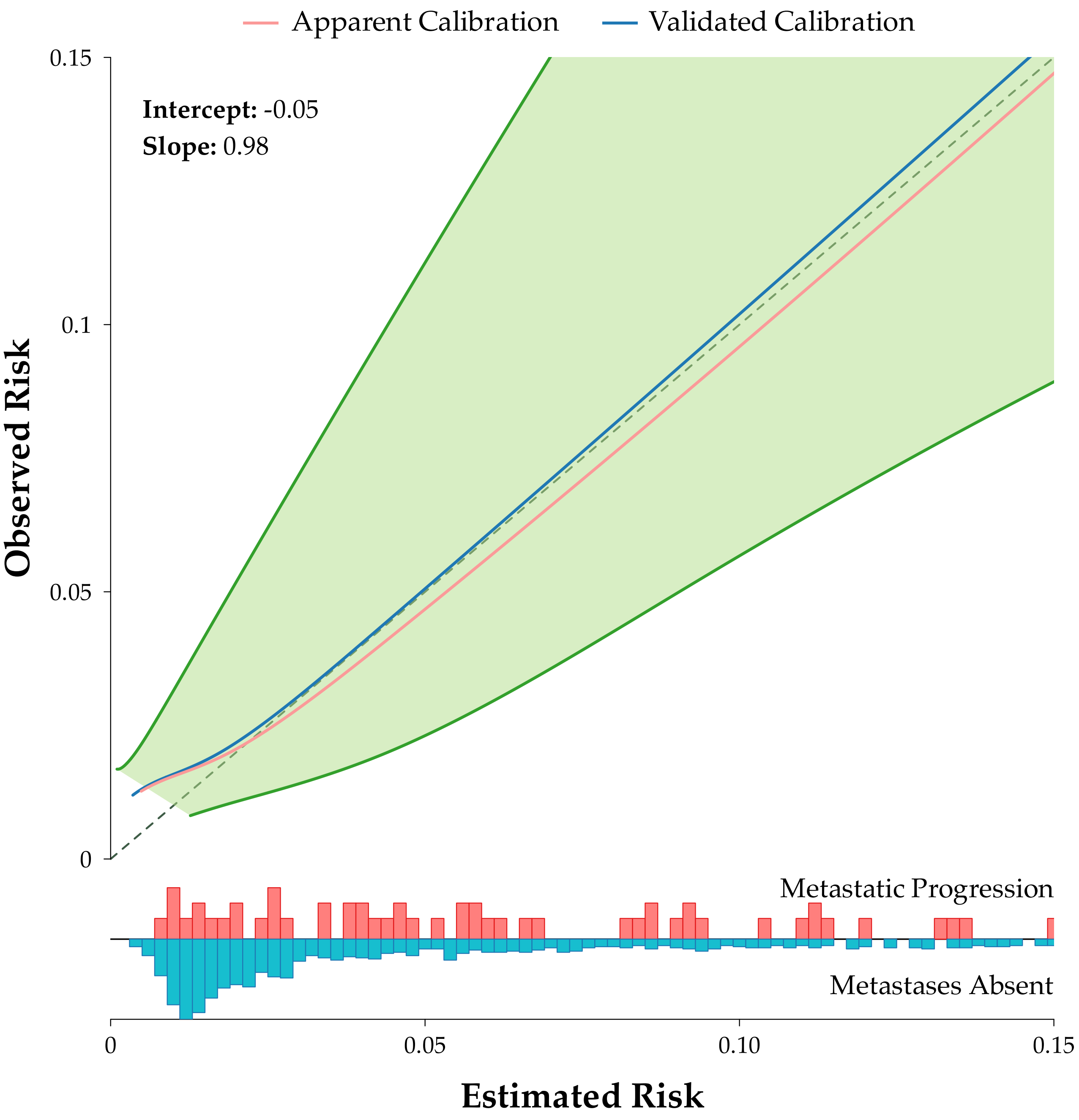

Figure S5: Zoomed calibration plot for metastatic prostate cancer (estimated risk range $0 - 0.15$). Apparent and validated (optimism-corrected) calibration curves are shown, with the 95% bootstrap confidence band. The lower confidence band is displayed for estimated risks 0.1% due to spline boundary instability at very low predicted risks.

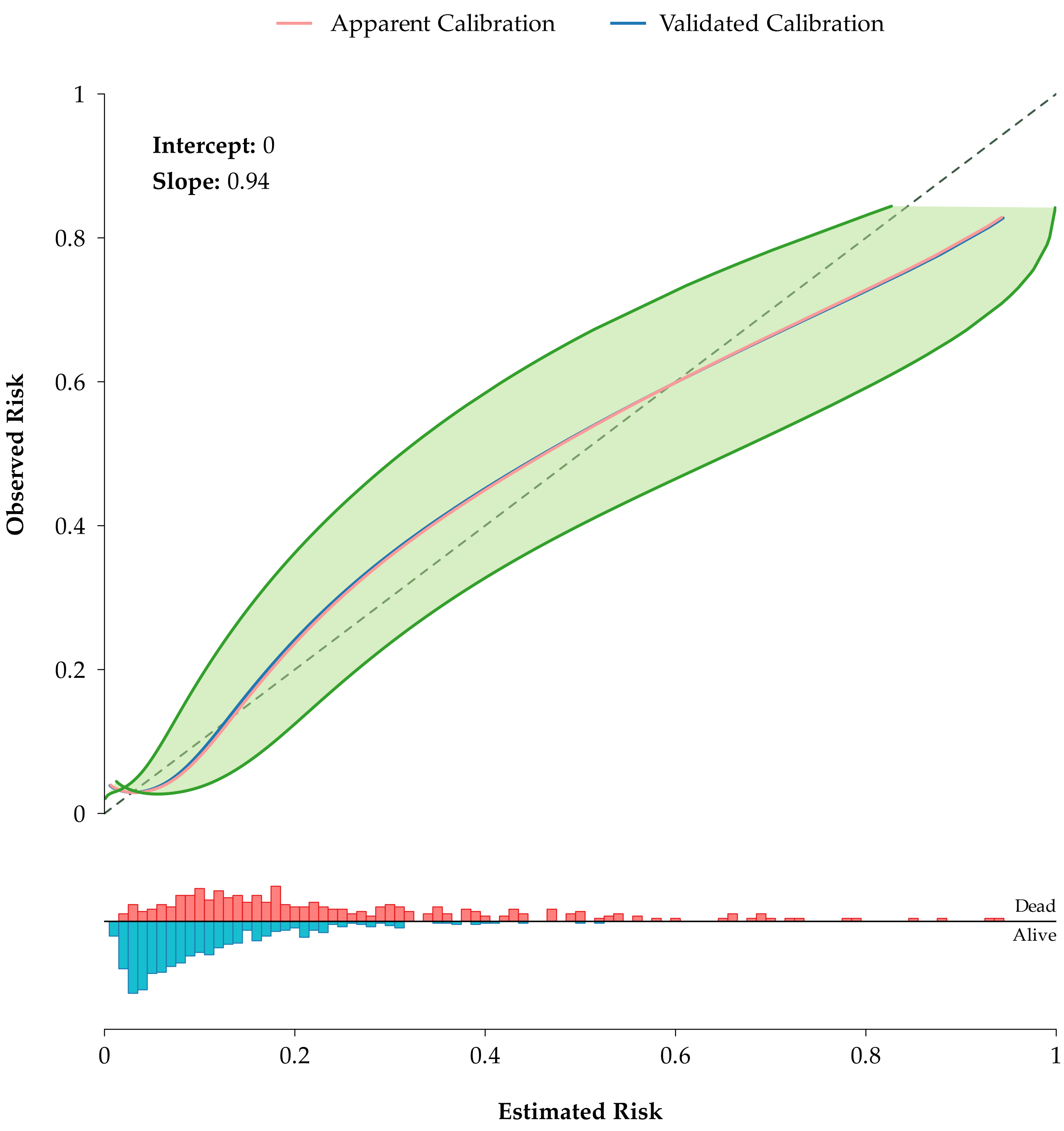

Figure S6: MSKCC calibration plot for overall mortality at five years post-diagnosis, following internal validation using bootstrap sampling (500 repetitions) and multiple imputation across 20 datasets. The "apparent" and "validated" lines represent the median calibration plots across the 20 imputed datasets. Confidence intervals are defined by the medians of the 2.5th and 97.5th percentiles across the 500 bootstrap samples for each imputed dataset. The histogram along the x-axis shows the distribution of risk estimates, stratified by deceased and surviving patients. MSKCC: Memorial Sloan Kettering Cancer Center.

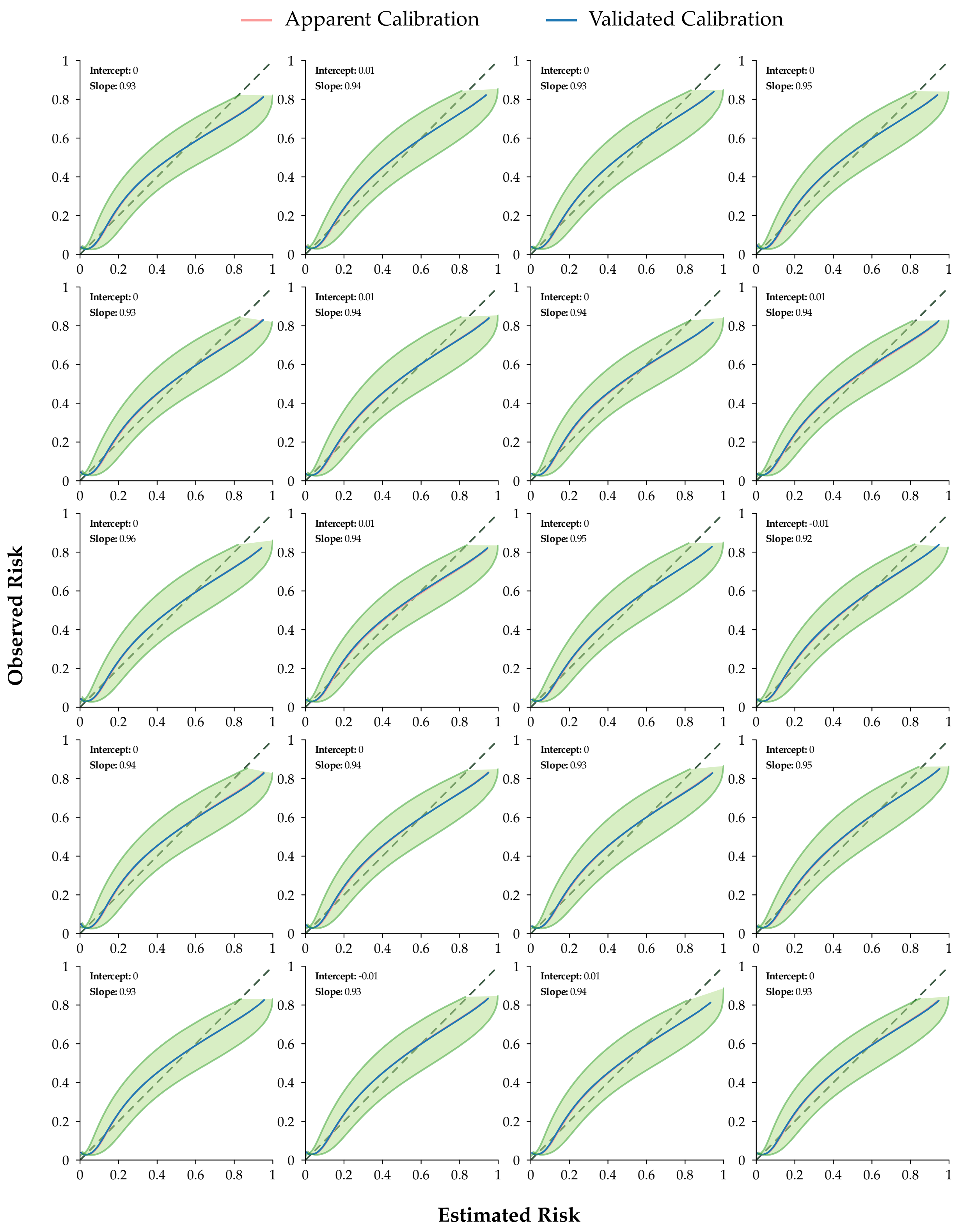

Figure S7: Apparent and internally validated calibration plots for MSKCC predicting overall mortality five years after diagnosis, for each imputed dataset. The green area represents the range between the 2.5th and 97.5th percentiles across the 500 bootstrap samples. Intercept and slope estimates for the outcome are reported for each imputed dataset. MSKCC: Memorial Sloan Kettering Cancer Centre.
